# Survey of collaboration supporting students who are deaf and hard of hearing

**DOI:** 10.1093/deafed/enae006

**Published:** 2024-02-26

**Authors:** Samantha J Gustafson, Elsa Newsome, Nicole Pilling, Emilee Segura

**Affiliations:** Department of Communication Sciences and Disorders, University of Utah, Salt Lake City, United States; Department of Communication Sciences and Disorders, University of Utah, Salt Lake City, United States; Department of Special Education, University of Utah, Salt Lake City, United States; Department of Special Education, University of Utah, Salt Lake City, United States

## Abstract

Collaboration between teachers of students who are deaf and hard of hearing (TSDHH) and educational audiologists is essential when developing successful, comprehensive service delivery plans for students who are deaf and hard of hearing. Despite the importance, little is known about how these two professions work together. This study sought to describe the current state of collaboration between educational audiologists and TSDHH and to explore the barriers and facilitators to this collaboration. Anonymous survey responses from 752 educational audiologists and TSDHH showed that collaboration is considered valuable and is occurring frequently, via a variety of formats and despite significant barriers. More research is needed to understand how efforts to minimize barriers to collaboration might improve the quality of collaboration and ultimately impact the success of student support.

School-age children who are deaf and hard of hearing (DHH) have a wide range of unique academic, linguistic, and social–emotional needs that are too complex to be met by a professional from a single discipline. These high-intensity needs require the services of an interdisciplinary team of professionals with specialized knowledge and skills in the area of hearing loss. Teachers of students who are DHH (TSDHH) and educational audiologists are two of the most essential service providers for school-age children who are DHH. With appropriate collaboration, they can meet students’ developmental and academic needs, ensure language and communication access, and provide education in the least restrictive environment (Educational Audiology Association [EAA], 2018). As such, collaboration between these professions is crucial in developing successful, comprehensive service delivery plans (National Association of State Directors of Special Education [NASDSE], 2018). Unfortunately, anecdotal reports suggest that current collaborative practices are unsatisfactory, with educational audiologists and TSDHH rarely collaborating and often performing duties outside their respective scopes of practice (Meyer, 2019). These less-than-ideal patterns of practice place children who are DHH at risk for gaps in service provision. Thus, there is a critical need to identify elements of successful partnerships and barriers to effective collaboration between educational audiologists and TSDHH.

Difficult to define, collaboration is typically described as “working together.” Cook and Friend (2010) describe a more nuanced understanding, where collaboration is about how individuals share their work—characterizing it by the voluntary nature and inclusion of mutual goals, shared responsibility for critical decisions, joint accountability for outcomes, and shared resources. Cloninger (2017) describes a collaborative team as “a group of professionals working together on the four major areas of educational programming – assessment, development of instructional goals, intervention, and evaluation – with the shared goal of supporting student and family valued life outcomes.” Each team member brings a unique set of skills, perspectives, and experiences to the team relationship, which—when shared—can provide collaborators with a more comprehensive understanding of each student’s needs.

This sharing should allow for more effective planning and implementation of instruction and services and, thus, improved student outcomes. Several large-scale studies have shown a positive association between levels of teacher collaboration and student achievement, even when controlling for student-level (e.g., gender, race, socio-economic status, prior achievement) and school-level variables (e.g., school size, proportion of minority students; Goddard et al., 2007, 2010; Ronfeldt et al., 2015). Additionally, collaboration between special and general education teachers has been identified in multiple studies as a key strategy used in schools with high-performing students in special education (Caron & McLaughlin, 2002; Huberman et al., 2012). Beyond student outcomes, professional collaboration among teachers is associated with job satisfaction and work commitment (Duyar et al., 2013; García Torres, 2019). Supported by this empirical evidence, professional education organizations, including the Council for Exceptional Children, actively advocate for and endorse the importance of collaborative practices in fostering effective educational outcomes for students with disabilities (McLeskey et al., 2017). Furthermore, the multi-disciplinary team approach mandated by the Individuals with Disabilities Education Act (IDEA) for the identification, evaluation, and provision of services for students with disabilities further emphasizes the recognized value of collaboration (*The Individuals with Disabilities Education Improvement Act of 2004.* 20 U.S.C, 2004).

The importance of collaboration among educational service providers who work specifically with children who are DHH cannot be understated. The NASDSE emphasizes collaboration in their educational service guidelines, stating that “education personnel who work with students who are DHH encompass a wide range of knowledge, skills and abilities to meet the variety of services and supports needed by each child. Collaboration among specialized instructional support personnel, families, students and communities is a key component to successful provision of services” (NASDSE, 2018, p. 93). Despite the importance of partnership between educational audiologists and TSDHH, little is known about the collaborative relationships between the disciplines.

Most of the limited research examining collaboration in support of children who are DHH has surveyed related service providers (e.g., educational audiologists, speech-language pathologists [SLPs]) and general special educators, not TSDHH specifically. For example, Knickelbein and Richburg investigated the extent to which educational audiologists were available for consultation and support to special education teachers and SLPs, all of whom reported to work with students who are DHH. Only 59% of special educators reported that they had access to an educational audiologist in some or all of their schools, and only 63% who had access to an educational audiologist reported moderately or extremely good collaboration (Knickelbein & Richburg, 2012). Of the SLPs surveyed, 61% reportedly had access to an audiologist in all or some of their schools and only 37% of them rated the strength of collaboration with educational audiologists as moderately or extremely good (Richburg & Knickelbein, 2011). This research suggests the presence of two issues: access to the educational audiologist and, when access is present, strength of the collaboration.

Only one study has documented the collaboration between TSDHH and audiologists. Page et al. (2018) surveyed school professionals who work with children who are DHH in preschool and primary school settings about their experiences serving children who are DHH. Their sample included mostly SLPs (*n* = 123) and TSDHH (*n* = 105), with a handful of other professionals (e.g., special educators, early childhood educators, SLP assistants). One of the survey questions asked how frequently they communicated with their student’s audiologist. Overall, providers at the preschool level reported more frequent communication with their student’s audiologist than those in a primary school setting. TSDHH were more likely to communicate with the audiologist (57%) than were the SLPs (22.3%). Of the TSDHH surveyed, 60% reported communicating with the audiologist daily, weekly, or monthly, while the rest reported communicating four or less times per year, if at all. Page and colleagues called for future research to identify barriers to communication between professionals and to understand how we can increase collaboration between providers who serve children who are DHH. It is important to note that Page and colleagues did not differentiate between educational audiologists and clinical audiologists for their survey. Therefore, the true state of collaboration between educational audiologists and TSDHH is currently unknown.

Although research has not yet examined barriers and facilitators to collaboration between educational audiologists and TSDHH, exploring what contributes to and obstructs collaboration has received some attention in other areas of the education system and has received considerable attention in the medical community. Studies examining collaboration of educators have identified shared experiences, valuing and respecting the expertise of team members, trust, and common goals as facilitators (Ainscow, 2016; McLeskey et al., 2017; Stein & Short, 2001). Inadequate resources (e.g., time, personnel) and lack of clear roles and responsibilities of team members are reported to act as barriers to collaboration (Johnson et al., 2014; Stein & Short, 2001). Reports of interprofessional collaboration in the medical fields are generally consistent with the education literature but include several additional barriers and facilitators (McInnes et al., 2015; Wei et al., 2022). Additional facilitators include effective and open communication, shared work location, power sharing, and well-identified protocols and standards. Stress/burnout, staffing shortages, lack of training in collaborative practice, and resistance of administration/management have been identified as barriers to collaboration. Given the consistencies across the education and medical communities, it seems reasonable that educational audiologists and TSDHH might report similar barriers and facilitators to collaboration.

The purpose of this study was to describe the current state of collaboration between educational audiologists and TSDHH. We conducted a survey to improve our understanding of the accessibility and use of collaborative practices between these two professions. Based on previous research and anecdotal reports (Meyer, 2019; Page et al., 2018), we expected that not all respondents would report that they collaborated with the other professional. A secondary purpose of this study was to explore the barriers and facilitators to collaboration, as reported by educational audiologists and TSDHH.

## Method

### Survey development

The survey was originally developed by a research audiologist and a TSDHH. Questions were developed to gather information about (1) the respondent; (2) the frequency, purpose, and method of collaboration; and (3) the perceived barriers to and facilitators of collaboration. The initial draft of the survey received extensive feedback from three experts in educational audiology and deaf education. Following their feedback, a pilot test was conducted to assess feasibility and ensure that the survey was organized and functioning properly. Twelve professionals that represented the target population (4 audiologists and 8 TSDHH) were asked to complete the pilot survey and provide feedback. Pilot data identified the need to clarify confusing questions, adjust rating scale options, and add additional items. The final survey included 110 items; however, because some items were specific to each profession, not all respondents answered all 110 items. The survey was expected to take the respondents 15–20 min to complete and can be found in our Supplemental Materials.

### Study procedures

The survey was housed in an electronic survey software: REDCap (Harris et al., 2009, 2019). Educational audiologists and TSDHH were notified of the opportunity to participate in the survey through multiple methods including email list-serves of professional organizations, social media posts on Instagram and Facebook groups dedicated to audiology and education of students who are DHH, and printed flyers mailed to schools for the Deaf around the United States. In attempts to include participants from other English-speaking countries, a flyer was also emailed to deaf education schools in England, Australia, and Ireland. Participants were able to click on a direct link or scan a QR code to access the web-based survey. The online survey was live from November 1, 2021, to March 31, 2022. All survey responses were anonymous. Respondents had the opportunity to be entered into a drawing for one of three $100 gift cards. This study and all procedures were approved by the university’s Institutional Review Board.

### Data analysis

Quantitative survey data were summarized by two researchers. Four researchers, including the two who performed quantitative summary, analyzed the qualitative data. The qualitative data analysis process was a modified version of that used by Richards and Hemphill (2018). Specifically, a collaborative qualitative analysis (CQA) was performed for questions with *>*100 written responses. Questions with <100 responses were analyzed by two researchers (one audiologist and one TSDHH). The first step of CQA was open coding. Each researcher read 10 different responses and noted common themes and main ideas. One researcher used these notes to create a preliminary codebook. During step two, the entire team met to discuss and agree upon the preliminary codebook. Step three included piloting of the preliminary codebook—where all members of the team read and coded the same 25 responses. In step four, the codebook was amended as needed following a team discussion of the preliminary codebook piloting. Split coding was used during the final coding process, where the responses to each question were split across the team members to be coded. The sixth and final step was a group discussion to resolve disagreements and finalize themes and main ideas.

## Results

### Survey respondents

Seven hundred fifty-seven surveys were initiated. Of these, 542 were fully completed and 215 were partially completed. Partial survey responses were included in data analyses. The first question of the survey asked participants to report if they were licensed to practice audiology or teach students who are DHH. Three respondents answered “no” and one did not answer—these data were removed from the sample. Of the remaining 753 survey responses, 34.3% (258) were from licensed audiologists, 63.2% (476) were from licensed TSDHH, and 2.4% (18) were from respondents who reported to be licensed as an audiologist and a TSDHH. One additional respondent did not disclose their licensure type before exiting the survey—their data were removed from the sample. Of those that reported licensure in both professions, respondents were asked to choose one professional identity through which they would answer the survey questions: 6 chose to respond as an audiologist and 12 chose to respond as a TSDHH. Due to the anonymity of the survey, we are unable to determine if the respondents indicating dual licensure took the survey one or two times (once as an audiologist and once as a TSDHH). Unless otherwise reported, all percentages were calculated using 264 (total number of responses from audiologists) and 488 (total number of responses from TSDHH).

Information about employment and preparation from respondents is provided in Table 1. In addition to country of practice, respondents were asked to report their years of experience in the field. Responses were received from professionals in multiple countries, with >90% reporting to practice in the United States. The distribution of professional experience was similar between audiologists and TSDHH, with nearly 60% practicing for over 10 years. Respondents used a sliding scale (0–40) to estimate the number of hours per week that they dedicate to educational audiology (for audiologists) or to serving children who are DHH (for TSDHH). Their estimation was instructed to include travel time, testing, training, consulting, report writing, etc. The majority of both audiologists and TSDHH reported spending >30 hr/week in their respective roles. Respondents were also asked whether they received specialized training in their graduate program that might have prepared them to work with school-age children who are DHH, namely, training in audiology for TSDHH, in educational audiology for audiologists, and in collaboration for both professions. TSDHH training programs attended by respondents were more likely to include training in audiology (84.8%) than audiology training programs to include training in educational audiology (60.6%). Training in collaborating with other professionals was included for 70% of the TSDHH and 59.1% of audiologists.

**Table 1:** Demographic and professional information about survey respondents.

	EdAuds	TSDHH
	(*n* = 264)	(*n* = 488)
Country of practice: *n (%)*		
Argentina	1 (.36%)	–
Australia	–	9 (1.84%)
Canada	8 (3.03%)	20 (4.10%)
India	–	2 (.41%)
New Zealand	–	1 (.20%)
United Kingdom	3 (1.14%)	16 (3.28%)
United States	250 (94.7%)	440 (90.2%)
Professional experience: *n* (%)		
Less than 1 year	7 (2.65%)	17 (3.5%)
1–5 years	49 (18.6%)	92 (18.9%)
6–10 years	47 (17.8%)	92 (18.9%)
11–20 years	51 (19.3%)	142 (29.1%)
20+ years	107 (4.5%)	145 (29.7%)
Estimated hours per week for educational audiology (EdAuds)/serving children who are DHH (TSDHH): *n* (%)^a^		
1–10 hr	16 (6.35%)	7 (1.53%)
11–20 hr	27 (10.7%)	44 (9.63%)
21–30 hr	33 (13.1%)	67 (14.6%)
31–40 hr	176 (69.8%)	339 (74.2%)
Graduate training: *n* (%)^b^		
Received training in audiology	–	414 (84.8%)
Received training in educational audiology	160 (60.6%)	–
Received training in collaboration^a^	156 (59.1%)	340 (7.0%)

*Note*. En dashes indicate data not obtained/reported. TSDHH = teacher of students who are deaf/hard of hearing; EdAud = educational audiologist.

^a^Two hundred fifty-two audiologists and 457 TSDHH provided responses to this question.

^b^Only 486 respondents who identified as TSDHH answered this question.

Respondents were employed in a variety of work settings, some in multiple settings. Of the audiology respondents, 70.1% reported that they were employed directly by the educational entity, 22.7% reported being contracted by the educational entity, and 3.4% reported that they were employed by a different classification (e.g., consultant, higher education, school district that contracts with other school districts, educational outreach through a hospital). Of the TSDHH, 56.4% worked in general education settings as an itinerant teacher, 20.7% worked in general education settings in a self-contained or resource classroom, 17.2% worked in a day school/program for children who are DHH, 13.1% worked in an early intervention program, 9.2% worked in a residential school, and 6.4% reported a different classification (e.g., consultant, work in multiple school districts, member of the school board, higher education).

### Collaborative practice patterns

Because collaboration cannot occur without access to the other professional, we first asked whether or not the audiologist respondent had access to a TSDHH and the TSDHH respondent to an educational audiologist. Of the 250 audiologists responding to this question, 98.8% reported access to a TSDHH and 1.2% were unsure. Of the 446 TSDHH responding to this question, 80.7% reported access to an educational audiologist, 17.7% reported no access, and 1.6% were unsure. TSDHH who reported no access to an educational audiologist were asked to explain why they did not have access in an open-response format. The main themes emerging from these responses were that their lack of access was due to the (1) lack of personnel (e.g., the county/district does not have an educational audiologist due to rural area or small student population, the previous educational audiologist retired and no one has applied to fill the role), (2), absence of district support (e.g., not enough district resources/funding, district’s lack of understanding the difference between or need for both TSDHH and educational audiologists), and (3) use of alternative support (e.g., TSDHH connects directly with the child’s private/clinical audiologist, TSDHH contacts an audiologist friend rather than the district’s educational audiologist).

For respondents who reported access to the other professional, 100% (225/225) of audiologists and 98.2% (328/334) of TSDHH report collaborating. The frequency with which these respondents reported collaborating is shown in Table 2. The majority of these respondents from both professions reported collaborating either daily (55% of audiologists, 12% of TSDHH) or weekly (39% of audiologists, 48% of TSDHH). To estimate the time spent collaborating synchronously, where both professionals are interacting in real time, we asked these respondents to estimate the amount of time, in a typical week, that they collaborate with the other professional via phone, face to face, or videoconferencing. Their estimations were entered using a sliding scale that was anchored in minutes or hours, based on their prior response of daily/weekly/monthly collaboration. Using these data, and the assumption that there are 5 days in a work week, 4 weeks in a month, 18 weeks in a semester, and 36 weeks in a school year, the number of hours per week spent synchronously collaborating was estimated for each respondent (audiologists *n* = 224, TSDHH *n* = 326). Audiologists reported to spend more hours per week (*M* = 6.74, *SD* = 7.23) synchronously collaborating than TSDHH (*M* = 1.91, *SD* = 3.84; *t*(548) = 10.158, *p* < .001). Audiologists’ estimates ranged from 0 to 33.3 hr/week while TSDHH estimates ranged from 0 to 28.67 hr/week. Three audiologists (1.33%) and 27 TSDHH (8.28%) reported that they do not spend any time in a typical week synchronously collaborating. Because collaboration can also occur asynchronously—via email and text messaging—we asked respondents to indicate which methods of collaboration they use. The frequency with which respondents reported using each of the varying collaboration methods is also reported in Table 2. Email was the most common method of collaboration for both groups, followed by one-on-one meetings in person, texting, and then phone calls. Those that selected “other” when asked about methods of collaboration noted the use of cloud-based sharing platforms, joint testing, social media platforms, and instant messaging. Finally, we asked respondents to estimate, for a typical week, how many points of contact (e.g., individual interactions or communications via email, phone call, text message, meeting) they made with professionals from the other discipline. Based on their prior response of daily/weekly/monthly collaboration, respondents were asked to estimate how many points of contact they had in a typical day/week/month from the seven choices provided (1, 2, 3, 4, 5–7, 8–10, and 11+). In general, audiologists reported having more points of contact per week with the TSDHH than TSDHH reported for their points of contact with educational audiologists.

**Table 2:** Collaboration details provided by respondents.

	EdAuds	TSDHH
	(*n* = 225)	(*n* = 328)
Frequency of collaboration: *n* (%)		
Daily	121 (54.8%)	40 (12.0%)
Weekly	87 (38.7%)	159 (47.6%)
Monthly	15 (6.67%)	89 (26.7%)
Once per semester	1 (.44%)	22 (6.59%)
Annually	1 (.44%)	17 (5.09%)
Method of collaboration: *n* (%)^a^		
Phone	189 (84.4%)	206 (63.4%)
In person (one-on-one)	202 (90.2%)	224 (75.1%)
In person (team)	165 (73.7%)	141 (43.4%)
Email	219 (97.8%)	298 (91.7%)
Texting	191 (85.3%)	207 (63.7%)
Video conferencing	135 (60.3%)	91 (28.0%)
Other (provide details)	10 (4.46%)	2 (.615%)
Estimated number of contact points per week: *n* (%)^a^		
Less than 1 point of contact	7 (3.11%)	98 (30.1%)
1–2 points of contact	21 (9.33%)	86 (26.4%)
>2–5 points of contact	25 (11.1%)	69 (21.2%)
>5–10 points of contact	54 (24.0%)	44 (13.5%)
>10–20 points of contact	55 (24.4%)	17 (5.21%)
20+ points of contact	63 (28.0%)	12 (3.68%)

*Note*. TSDHH = teacher of students who are deaf/hard of hearing; EdAud = educational audiologist.

^a^Two hundred twenty-four audiologists and 325 TSDHH provided responses to this question.

Respondents were asked to describe the purpose of their collaboration by selecting responses from a list. The frequency of responses for each listed purpose for collaboration can be found in Table 3. The vast majority of audiologists noted collaborating with a TSDHH for a number of reasons—primarily discussion of evaluations and educational programming/accommodations (96.0%), troubleshooting equipment (90.2%), addressing questions/concerns regarding student progress (88.4%), and developing IEP/504 plans (76.3%). Audiologist respondents who indicated that they collaborate for other purposes noted collaborating to educate/support families, provide self-advocacy instruction to students, schedule appointments for audiologic evaluations, and discuss the impact of hearing and hearing assistive technology on a student’s access to instruction. Most of the TSDHH reported collaborating primarily to troubleshoot equipment (87.7%). TSDHH respondents indicating collaboration for purposes other than the options provided in the survey described collaborating for functional listening evaluations, staff training/professional development, ordering/installing/managing remote microphone systems and other equipment, and parent education.

**Table 3:** Purpose of collaboration as reported by respondents.

EdAuds (*n* = 224)	
Discuss evaluations and educational programming/accommodations	215 (96.0%)
Troubleshooting equipment	202 (90.2%)
Address questions/concerns re: student progress	198 (88.4%)
IEP/504 development	171 (76.3%)
Provide training to TSDHH	132 (58.9%)
Other (provide details)	20 (8.9%)
TSDHH (*n* = 324)	
Troubleshooting equipment	284 (87.7%)
Remote microphone system management	191 (59.0%)
Educational programming/accommodations	180 (55.6%)
Address questions and concerns regarding student progress	158 (48.8%)
Coordinating/interpreting audiologic testing	153 (47.2%)
IEP/504 development	152 (46.9%)
Other (provide details)	33 (10.2%)

*Note*. TSDHH = teacher of students who are deaf/hard of hearing; EdAud = educational audiologist.

The respondents who reported to have access to their professional counterpart and to currently collaborate with them (audiologists *n* = 201, TSDHH *n* = 275) were asked a series of questions requiring them to reflect on their perceptions of the value, strength, and benefits of collaboration between educational audiologists and TSDHH. Questions and responses are shown in Table 4. Respondents from both professions overwhelmingly reported that collaboration between educational audiologists and TSDHH is valuable, beneficial for the students, and makes their jobs easier. All respondents were provided with the opportunity to elaborate on the value of collaboration between educational audiologists and TSDHH—518 respondents provided free-text responses (audiologists *n* = 185, TSDHH *n* = 333). Table 5 shows the four main themes emerging from these responses as well as example responses for each theme. One hundred and eighty-six respondents provided free-text responses including an example of a time when collaboration benefited them as a professional or benefitted their student(s). In these examples, educational audiologists primarily noted situations when the TSDHH caught equipment malfunction or re-programming needs or when they worked together to assess/ensure auditory access in the classroom. Educational audiologists also reported that they collaborated to ensure/monitor assistive technology use and they teamed up to troubleshoot equipment and support the student’s family. TSDHH examples primarily included working together to learn how to use, manage, troubleshoot, and update hearing assistive technology and to support families and students with special listening needs (e.g., auditory processing disorder, auditory neuropathy spectrum disorder, additional disabilities). TSDHH also reported that they collaborated to improve their understanding of audiograms and hearing loss and to provide students with better access to the auditory environment (and thus, curriculum). Among the many responses to this question, many respondents simply took the opportunity to express enthusiasm for their collaboration with the other professional.

**Table 4:** Perceptions of collaboration from educational audiologists (EdAuds) and teachers of students who are deaf/hard of hearing (TSDHH) who report to currently collaborate.

How valuable do you consider collaboration between educational audiologists and TSDHH?					
	Extremely valuable	Quite valuable	Moderately valuable	Slightly valuable	Not at all valuable
EdAud	87.10%	11.40%	1.50%	0.00%	0.00%
TSDHH	69.10%	21.80%	6.90%	2.20%	0.00%
Please rate the strength of your collaboration with the educational audiologist/TSDHH.					
	Excellent	Very good	Good	Fair	Poor
EdAud	55.20%	30.80%	11.40%	2.50%	0%%
TSDHH	54.50%	26.50%	1.50%	6.20%	2.20%
My students benefit from my collaboration with the educational audiologist/TSDHH.					
	Strongly agree	Somewhat agree	Neither agree nor disagree	Somewhat disagree	Strongly disagree
EdAud	9.50%	6.50%	0.50%	0%	2.50%
TSDHH	77.10%	15.30%	4.40%	1.10%	2.20%
Collaborating with the educational audiologist/TSDHH makes my job easier.					
	Strongly agree	Somewhat agree	Neither agree nor disagree	Somewhat disagree	Strongly disagree
EdAud	78.60%	15.90%	2.50%	0.50%	2.50%
TSDHH	74.20%	17.50%	3.60%	2.50%	2.20%
I feel confident in my ability to collaborate with the educational audiologist/TSDHH.					
	Strongly agree	Somewhat agree	Neither agree nor disagree	Somewhat disagree	Strongly disagree
EdAud	77.10%	18.40%	1.50%	0.50%	2.50%
TSDHH	80.70%	12.00%	2.90%	1.80%	2.50%

*Note*. Data obtained from 201 EdAuds and 275 TSDHH.

**Table 5:** Reasons collaboration is valuable from educational audiologists and teachers for students who are deaf/hard of hearing (TSDHH)

Theme	%(*n*)	Example
Collaboration is valuable because the roles, skill-sets, and perspectives of each professional are unique	3.0% (156)	“We have different skills sets and training that are equally beneficial to the student. We need both perspectives to assure the student has the access to everything they need to be successful.”– Educational Audiologist
Collaboration increases student success, allowing each professional to better meet the students’ needs by providing better services	18.15% (94)	“The audiologist and TSDHH collaboration ensures that the student’s needs are being met and accommodations are in place to give the student equal access to curriculum as their hearing peers”—TSDHH
Collaboration is valuable because the TSDHH sees the student/family more frequently than the educational audiologist	11.0% (57)	“[TSDHH] often get to spend more time with the students than I do, so they let me know about equipment or self advocacy issues, and help guide recommendations based on accurate student needs and student reports/concerns.” – Educational Audiologist
Collaboration allows them to continue learning from the other professional	3.67% (19)	“I have learned so much about my students’ equipment from our contracted audiologist. I am able to troubleshoot and resolve many issues that would take much longer if we had to wait for the audiologist to come. She is the provider for many of our students and has insights that we would otherwise miss. She is a great advocate for them.”—TSDHH

The majority of respondents reported confidence in their ability to collaborate and rated the strength of their collaboration as “Excellent” or “Very Good.” Notably, less than 10% of respondents reported a lack of confidence in their ability to collaborate. Respondents who reported not to have access to the other profession were also asked to rate the value of collaboration between educational audiologists and TSDHH. Only one audiologist who reported no access to a TSDHH answered this question—reporting collaboration to be moderately valuable. The distribution of responses from the 62 TSDHH without access to an educational audiologist was similar to that of TSDHH who did have access (Extremely valuable: 56%; Quite valuable: 29%; Moderately valuable: 9.7%, Slightly valuable: 3.2%; Not at all valuable: 1.6%). Seven TSDHH who reported to have access to an educational audiologist but that they did not collaborate with them also answered this question. As might be expected, their responses indicated less perceived value (Extremely valuable: 28.6%; Quite valuable: 0%; Moderately valuable: 57.1%, Slightly valuable: 14.3%; Not at all valuable: 0%).

Because this survey was completed during the COVID-19 pandemic, we asked a brief series of questions about how the COVID-19 pandemic affected collaboration between educational audiologists and TSDHH. Respondents (audiologists *n* = 208, TSDHH *n* = 302) provided free-text responses describing how their collaboration with the educational audiologist/TSDHH was different during the remote/hybrid learning period of the pandemic. Three themes emerged from these responses. The first theme was a discussion about the effect of remote/hybrid learning on the frequency of collaboration (audiologists *n* = 68, TSDHH *n* = 126). TSDHH respondents primarily described either no change or less collaboration (Less—53.2%, More—7.1%, No change—4.5%) while educational audiologists described a broad distribution of the effects that the pandemic had on the frequency of collaboration (Less—41.2%, More—2.6%, No change—38.2%). The second theme encompassed notes about how respondents collaborated during remote/hybrid learning. Respondents from both professions described a lack of in-person visits/meetings, that meetings were conducted virtually, and that asynchronous collaboration (e.g., text messages, emails) were more common. The third theme that emerged from these responses revolved around a shift in focus of collaboration toward technology and software required for student support—including connectivity and troubleshooting. Respondents from both professions reported a significant increase. When asked if they would revert (or have already) to their pre-pandemic ways of collaborating with the educational audiologist/TSDHH once in-person learning has resumed, TSDHH were more likely to answer “Yes” than educational audiologists (TSDHH: Yes—75.9%, No—8.36%, Somewhat—15.8%; educational audiologists: Yes—51.6%, No—12.1%, Somewhat—36.3%). Respondents who answered “No” or “Somewhat” to the previous question were asked to describe why they would not return to their pre-pandemic ways of collaboration. Both TSDHH and educational audiologists reported that they would continue the virtual meeting format because it is more convenient, productive, and flexible—offering better access to collaboration than in-person methods.

### Barriers and facilitators of collaboration

**Figure 1:**
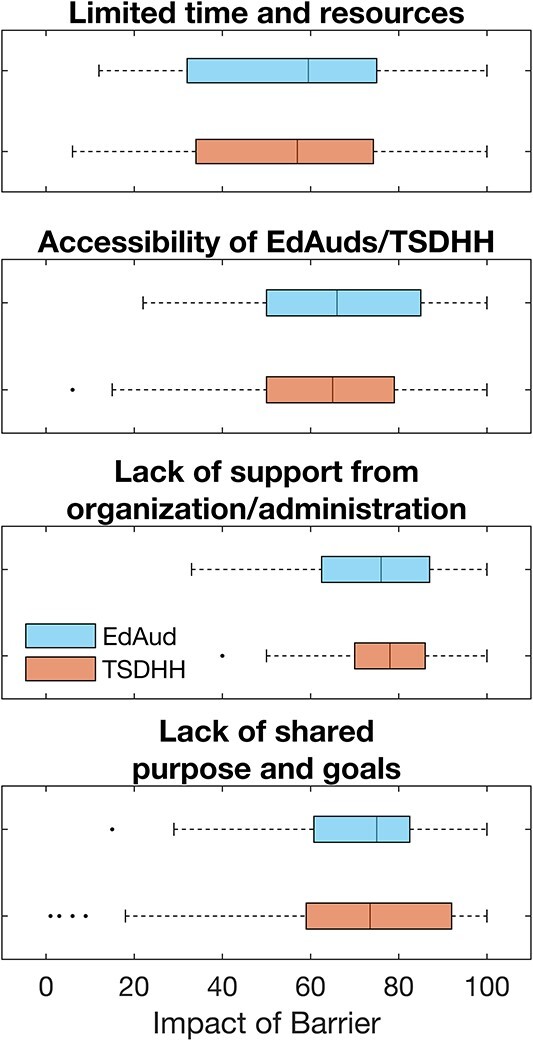
Impact of barrier on collaboration (0 = not at all; 100 = substantial) reported by educational audiologists (EdAuds) and teachers of students who are deaf and hard of hearing (TSDHH).

All respondents were asked to select items from a list that they considered barriers to collaboration with the educational audiologist/TSDHH. For barriers that were selected, respondents were also asked to provide an estimation of the impact that this challenge had on their collaboration using a sliding scale anchored in “not at all” and “substantial.” Of the responses to this question (audiologist *n* = 202, TSDHH *n* = 344), 36.6% of audiologists and 37.5% of TSDHH reported that they did not have any barriers to collaboration. The most prominent barrier selected was “Limited time and resources” (44.6% of audiologists, 45.1% of TSDHH) followed by “Accessibility of the educational audiologist or TSDHH” (12.4% of audiologists, 24.4% of TSDHH). “Lack of support from my organization/administrator” was selected by 17.8% of audiologists and 11.6% of TSDHH, and “Lack of shared purpose and goals” was selected by 10.9% of audiologists and 6.4% of TSDHH. Figure 1 shows the reported impact of these barriers on respondents’ ability to collaborate, rated on a scale from 0 (*not at all*) to 100 (*substantial*). Ratings of impact varied widely across all respondents; however, median values for both professions fell above 50 for “Limited time and resources” and above 60 for the remaining three barriers. Fifty respondents (audiologist *n* = 23, TSDHH *n* = 27) reported that they experienced a challenge in collaborating that was not listed. Analysis of these open responses from audiologists revealed that differing opinions between the respondent and the TSDHH and instances in which the TSDHH operates outside their scope of practice were barriers to collaboration. TSDHH responding to this question described conflicts due to lack of trust and a feeling that audiologists did not understand the educational impact of hearing loss. Respondents from both professions described barriers that included confusion of roles and responsibilities, poor communication/difficult person to work with, and lack of desire to collaborate and/or limited understanding of the benefit of collaboration.

In the final open-response question of the survey (“*If there is anything else you would like to share about your collaboration (or lack of collaboration) with the educational audiologist/TSDHH, please provide those details below”)*, 31 participants mentioned specific barriers to collaboration. Several echoed the barriers discussed above (e.g., limited access to the other professional, lack of financial resources, lack of understanding of the other profession’s roles/responsibilities). Other responses included system-level challenges (e.g., district requirements, limited opportunities), difficulty recruiting/retaining personnel, and lack of adequate training. Notably, in these responses, limited access to the other professional did not simply mean that there was no one in that position. One TSDHH respondent noted, “*Our district contracts with another district that has a multi-district DHH program. The educational audiologists serve several districts in the area so sometimes they are not immediately available*.” One audiologist respondent described the complex interaction between systems-level challenges and understanding roles/responsibilities: “*I am aware of [TSDHH]’s in our state (and around the country) that function outside of their scope of practice (e.g., select and fit [hearing assistive technology]) because their [Special Education] Directors told them to, and they’re not aware that they should be advocating for educational audiology services*.”

All respondents who reported that they collaborated were presented with a list of strategies and asked to identify the ones that they found have been successful in fostering collaboration. The most prominent facilitator for successful collaboration for audiologists (*n* = 202) and TSDHH (*n* = 275) was forming and maintaining a relationship of mutual trust and respect (91.9% of audiologists, 92% of TSDHH). Respondents from both professions also indicated other facilitators of success, albeit at lesser frequency. Having an established time in their schedule for collaboration was considered a facilitator for 44.1% of audiologists and 40% of TSDHH. Jointly establishing protocols and procedures that clearly delineate roles and responsibilities to ensure student success was selected by 48% of audiologists and 38.9% of TSDHH. Seeking out mutual professional growth opportunities and training that addresses relevant challenges was selected by 5.5% of audiologists and 41.8% of TSDHH. Twenty-six respondents selected “Other” and provided strategies that they found successful in fostering collaboration in their own words. Audiologists reported shared office space as a strategy facilitating collaboration. Respondents from both professions noted that frequent communication (i.e., keeping the TSDHH updated on all correspondence as the case manager) and time spent outside of the work environment both facilitated collaboration.

Respondents were also asked if there was anything that the survey failed to capture that could improve the strength of their collaboration. TSDHH respondents mentioned the importance of trusting or deferring to the other professional when appropriate. Audiology respondents reported feeling that more auditory/oral, audiology, and technology training is needed for TSDHH and that everyone would benefit from a better-defined scope of practice for TSDHH. Respondents from both professions shared many consistent thoughts on what could improve collaboration. Namely, they noted that improved administrative support (including scheduled time for collaboration) and understanding of educational audiologists and TSDHH roles would be beneficial for collaboration. Similarly, respondents indicated that collaboration would be more successful if everyone understood each profession’s scope of practice. Having more training specific to collaboration between educational audiologists and TSDHH was noted, as were joint protocols for responsibilities that are shared by both professions. Finally, respondents from both fields noted that increasing the number of qualified personnel in both professions would improve the strength of collaboration. In the final open-response question of the survey, 21 respondents mentioned specific facilitators to collaboration. These responses mostly echoed those noted above (e.g., respectful relationship between professionals, open communication, administrative support); however, several respondents also noted that having a consistent person to contact for their whole caseload facilitated collaboration and that having an agreed-upon referral/intake process was helpful in collaborating.

## Discussion

This study sought to improve our understanding of the accessibility and use of collaborative practices between educational audiologists and TSDHH. To our knowledge, this is the first study to directly evaluate collaboration between educational audiologists and TSDHH. Our results indicate that collaboration is considered valuable and is occurring frequently despite significant barriers.

Because collaboration cannot occur without access to the other professional, in addition to examining rates and perceived value of collaboration, we asked respondents to disclose if they had access to their professional counterpart. Our results show that nearly all educational audiologists represented here have access to a TSDHH and that approximately 80% of TSDHH have access to an educational audiologist. Previous research examining access to educational audiologists reported that only 60% of special education teachers had access (Knickelbein & Richburg, 2012). It is difficult to determine if the increased access to educational audiologists found in our study reflects the specificity of our special education respondents (i.e., TSDHH) or represents an improvement in access to educational audiologists over the last decade.

For respondents with access to the other type of professional, a remarkably high amount of collaboration was reported, with 100% of audiologists and 98% of TSDHH reporting to collaborate with the other professional. Respondents from both professions overwhelmingly reported collaboration between educational audiologists and TSDHH to be valuable, to be beneficial for the students, and to make their jobs easier. Interestingly, the vast majority of TSDHH who do not currently have access to an educational audiologist reported that collaboration between educational audiologists and TSDHH was “quite valuable” or “extremely valuable.” This suggests that TSDHH who do not currently have access believe they would find value in having an educational audiologist to collaborate with.

A variety of methods, some synchronous and others asynchronous, were used for collaboration. Educational audiologists reported spending significantly more hours per week collaborating synchronously than TSDHH. The reason for this imbalance in collaboration time across professions is unclear. It is possible that educational audiologists serve multiple TSDHH, while individual TSDHH work with one educational audiologist for all students on their caseload. This would create more collaboration opportunities for the educational audiologist than the TSDHH. Further research is needed to improve our understanding of the distribution of time spent collaborating across these two professions.

### Facilitators and barriers to collaboration

Consistent with previous collaboration research, our results suggest that respecting and valuing team members, building trust, and having common goals is vital for successful collaboration. Although collaboration was reported to be valuable and occurring frequently, 63% of respondents identified barriers to this collaboration. Barriers identified by previous research (i.e., inadequate resources, lack of clear roles/responsibilities; Johnson et al., 2014; McInnes et al., 2015; Stein & Short, 2001; Wei et al., 2022) were also described by our sample. Limited time and resources were the most commonly reported barrier by both types of professionals and had a widely varying impact on their collaboration (see Figure 1). Lack of organization/administration support was the most significantly impactful barrier reported by both professions. Interestingly, nearly 20% of respondents reported that accessibility of the other professional was a barrier even though only 11% of respondents reported having no access to the other professional. This could suggest that simply having access to someone does not guarantee their availability throughout the entire school year. Respondents reporting additional barriers described confusion of roles and responsibilities, poor communication with the other professional, and limited understanding of the benefit of collaboration.

Lack of training in collaboration has been found to be a barrier for those in the medical community (Wei et al., 2022). Additionally, understanding and respecting team members’ expertise and roles, as well as knowledge gained in interdisciplinary training, are known facilitators. Our data showed that approximately 70% of TSDHH and 60% of audiologists received interdisciplinary training in their graduate programs. A promising trend was revealed when examining reported training across varying years of experience. Although only 51% and 57% of respondents with 20+ and 11–20 years of experience, respectively, reported training in collaboration, 80% of professionals with <10 years’ experience said they received training in collaboration. Future research is needed to directly examine the benefit of this apparent rise in interdisciplinary training on collaboration rates and student success.

Breaking down barriers to collaboration will likely require the efforts of multiple stakeholders and considerable resources. Results of this study provide actionable insights for educational audiologists, TSDHH, and administrators looking to enhance collaboration.

For example, collaboration might be enhanced, and student services improved, by providing educational audiologists and TSDHH with the opportunity to form and maintain relationships of mutual trust and respect. This could be accomplished by establishing protected time for collaboration, particularly to establish protocols and procedures together and engage in joint professional development. At a minimum, administrators should prioritize opportunities for the professionals to discuss and delineate their roles and responsibilities particularly in the context of their students’ service provision. One tool to help facilitate such discussion is the Shared and Suggested Roles of Educational Audiologists, Teachers of the Deaf and Hard of Hearing, and Speech-Language Pathologists checklist (Educational Audiology Association, 2018). This checklist describes the student services and support that must be considered by each team of professionals, including TSDHH, educational audiologists, and other service providers (as needed). This tool could help facilitate collaborative discussion between team members when developing and monitoring educational service plans and enables them to explicitly define their respective and shared roles and responsibilities. Future research is needed to determine if using tools such as this checklist can reduce the uncertainty surrounding roles and responsibilities between educational audiologists and TSDHH, which is considered a barrier to collaboration.

### Access to educational audiologists

Recall that, although every audiologist surveyed here reported having access to a TSDHH, nearly 18% of TSDHH reported that they did not have access to an educational audiologist. A major theme reported that explained the reason underlying this inaccessibility was the lack of an educational audiologist with whom to collaborate. This is consistent with the falling percentage of new audiologists choosing to work in educational settings (Council of Academic Programs in Communication Sciences and Disorders & American Speech-Langauage-Hearing Association, 2011, 2021). Given this growing shortage of educational audiologists, it is particularly concerning that our data show an uneven distribution of experience across educational audiologists represented in this survey. Specifically, 40% had more than 20 years of experience, 20% had between 11 and 20 years, and 40% had less than 10 years of experience. It is beyond the scope of this study to discuss this unequal distribution in depth; however, as the veteran educational audiologists begin to retire, it will be crucial to retain the newly trained educational audiologists and attract new audiologists to work in educational settings.

Exposure to educational audiology training at the graduate level could be one way to attract individuals to the field. Our data suggest that only 60% of educational audiologists surveyed here received training in educational audiology during their graduate programs. To understand how educational audiology training might have varied for audiologists who were trained across different time periods, we disaggregated the audiology training reports across years of experience. Only 44% of educational audiologists with 20+ years of experience reported having received educational audiology training in their graduate program. Training for audiologists with 11–20 years’ experience was slightly better, with 57% reporting that they received this training. Finally, 79% of educational audiologists with *<*10 years of experience reported that they received specific training in educational audiology. Although not a direct examination of how educational audiology training has been implemented over the years, these data appear to suggest that a greater number of audiology graduate training programs are including educational audiology content than ever before. Given the apparent increase in educational audiology training in graduate programs, it might be expected that more audiologists would choose to work in educational settings; however, this is not the case (as discussed in the previous paragraph). Future research should seek to understand the motivation behind the decision to work as an educational audiologist and the potential role that experiences in graduate training play in this decision.

### Limitations and future directions

In this study, we did not provide a definition of collaboration. We allowed the respondents to answer based on their personal definitions of collaboration. We acknowledge that there are different levels of collaboration. For example, the Assessment of Interprofessional Team Collaboration Scale (AITCS) measures a team’s varying attributes, or levels, of collaboration (Orchard et al., 2012), including (1) coordination (the ability to work together to achieve mutual goals), (2) cooperation (the ability to listen to and value the viewpoints of all team members and to contribute your own viewpoints), (3) shared decision making (all parties work together in exploring options and planning patients’ care in consultation with each other), and (4) partnership (creation of open and respectful relationships in which all members work equitably together to achieve shared outcomes). Using the AITCS could be useful in the classification of collaboration between educational audiologists and TDSHH, as it offers a framework for specifying that collaboration can be as simple as coordinating roles and responsibilities of each team member or as complex as meeting regularly to troubleshoot issues and to develop and evaluate educational service plans. Because respondents used their own definition of collaboration when completing this survey, it is unclear how varying interpretations of collaboration might have influenced the findings. Future research should be conducted using an established definition of collaboration to determine if the depth of collaboration between educational audiologists and TSDHH has an impact on success of student support.

We received a large number of survey responses (>750) from TSDHH and educational audiologists, primarily from professionals practicing in the United States. Respondents’ distribution of work setting and years of experience suggest that this sample is relatively representative of the broader, heterogeneous population of educational audiologists and TSDHH. Nevertheless, it is impossible for us to determine what percentage of the broader population of educational audiologists and TSDHH are represented in this sample. As such, results may not generalize to the broader population or to educational audiologists and TSDHH in other countries. Additionally, the survey did not control for or measure bias toward collaboration within the participants. The invitation to participate in this survey clearly asked educational audiologists and TSDHH to describe their experiences with collaboration. Although we cannot be certain, it is likely that this approach biased our results, as respondents were likely to be professionals who have knowledge of collaboration and engage in collaboration at some level. As such, rates and opinions of collaboration reported in this study may be higher and more positive than what might be present in the full population of educational audiologists and TSDHH.

Finally, future research is needed to address the paucity of evidence evaluating the effects of collaboration in education of students who are DHH. This is particularly true for TSDHH and educational audiologists, as those employed in both professions are being stretched beyond their capacity with large caseloads and students who have complex histories and educational needs. Our results suggest that it is not always feasible for these professionals to allocate sufficient time for collaboration. Future research demonstrating the efficacy of collaboration between TSDHH and educational audiologists on improving student outcomes could contribute to advocating for the additional resources required to support these professionals in their collaborative efforts. Considering the critical shortage of TSDHH and educational audiologists (American Speech-Language-Hearing Association, 2020; Luf, 2019), it would also be important to understand how collaboration influences job satisfaction and commitment. Results of this study suggest that TSDHH and educational audiologists overwhelmingly perceive collaboration to be beneficial for their students and to add value to their work. These data can serve as a foundation for future research that examine the direct effect of collaboration on student outcomes and job satisfaction.

## Funding

This work was supported by a Noel D Matkin Grant from the Educational Audiology Association to S.G. and N.P. and by the National Center for Advancing Translational Sciences of the National Institutes of Health (UL1TR002538). The data presented in this article will be shared on reasonable request to the corresponding author.

## References

[ref1] *The Individuals with Disabilities Education Improvement Act of 2004.* 20 U.S.C. (2004). § 1400 et seq.

[ref2] Ainscow, M. (2016). Collaboration as a strategy for promoting equity in education: Possibilities and barriers. Journal of Professional Capital and Community, 1(2), 1–20. 10.1108/JPCC-12-2015-0013.

[ref3] American Speech-Language-Hearing Association . (2020). Schools Survey Report: Trends in Educational Audiology 2010-2020. https://www-asha-org.ezproxy.lib.utah.edu/siteassets/surveys/2020-schools-survey-educational-audiology-trends.pdf.

[ref4] Caron, E. A., & McLaughlin, M. J. (2002). Indicators of Beacons of Excellence Schools: What Do They Tell Us About Collaborative Practices. Journal of Educational and Psychological Consultation, 13(4), 285–313. 10.1207/S1532768XJEPC1304_03.

[ref5] Cloninger, C. J. (2017). Designing Collaborative Educational Services. In Orelove F., Sobsey D., Silberman R. (Eds.). Educating Students with Severe and Multiple Disabilities: A Collaborative Approach (5th Ed., pp. 1–25). Baltimore, MD: Brookes Publishing.

[ref6] Cook, L., & Friend, M. (2010). The State of the Art of Collaboration on Behalf of Students With Disabilities. Journal of Educational and Psychological Consultation, 20(1), 1–8. 10.1080/10474410903535398.

[ref7] Council of Academic Programs in Communication Sciences and Disorders & American Speech-Langauage-Hearing Association . (2011). *2010–2011 Academic Year Communication Sciences and Disorders Aggregate Data Report*. https://www-asha-org.ezproxy.lib.utah.edu/Academic/HES/CSD-Education-Survey-Data-Reports/.

[ref8] Council of Academic Programs in Communication Sciences and Disorders & American Speech-Langauage-Hearing Association . (2021). *Communication Sciences and Disorders (CSD) Education Survey State Aggregate Data Report for Utah—2019-2020 Academic Year*.

[ref9] Duyar, I., Gumus, S., & Sukru Bellibas, M. (2013). Multilevel analysis of teacher work attitudes: The influence of principal leadership and teacher collaboration. International Journal of Educational Management, 27(7), 700–719. 10.1108/IJEM-09-2012-0107.

[ref10] Educational Audiology Association . (2018). Supporting Students who are Deaf and Hard of Hearing: Shared and Suggested Roles of Educational Audiologists, Teachers of the Deaf and Hard of Hearing, and Speech-Language Pathologists. http://www.edaud.org/position-stat/15-position-02-18.pdf.

[ref11] García Torres, D. (2019). Distributed leadership, professional collaboration, and teachers’ job satisfaction in U.S. schools. Teaching and Teacher Education, 79, 111–123. 10.1016/j.tate.2018.12.001.

[ref12] Goddard, Y. L., Goddard, R. D., & Tschannen-Moran, M. (2007). A Theoretical and Empirical Investigation of Teacher Collaboration for School Improvement and Student Achievement in Public Elementary Schools. Teachers College Record, 109(4), 877–896. 10.1177/016146810710900401.

[ref13] Goddard, Y. L., Neumerski, C. M., Goddard, R. D., Salloum, S. J., & Berebitsky, D. (2010). A Multilevel Exploratory Study of the Relationship Between Teachers’ Perceptions of Principals’ Instructional Support and Group Norms for Instruction in Elementary Schools. The Elementary School Journal, 111(2), 336–357. 10.1086/656303.

[ref14] Harris, P. A., Taylor, R., Minor, B. L., Elliott, V., Fernandez, M., O’Neal, L., McLeod, L., Delacqua, G., Delacqua, F., Kirby, J., & Duda, S. N. (2019). The REDCap consortium: Building an international community of software platform partners. Journal of Biomedical Informatics, 95, 103208. 10.1016/j.jbi.2019.103208. 31078660 PMC7254481

[ref15] Harris, P. A., Taylor, R., Thielke, R., Payne, J., Gonzalez, N., & Conde, J. G. (2009). Research electronic data capture (REDCap)—A metadata-driven methodology and workflow process for providing translational research informatics support. Journal of Biomedical Informatics, 42(2), 377–381. 10.1016/j.jbi.2008.08.010. 18929686 PMC2700030

[ref16] Huberman, M., Navo, M., & Parrish, T. (2012). Effective Practices in High Performing Districts Serving Students in Special Education. Journal of Special Education Leadership, 25(2), 59–71.

[ref17] Johnson, D. C., Cannon, L., Oyler, A., Seaton, J., Smiley, D., & Spangler, C. (2014). Shift happens: Evolving practices in school-based audiology. Journal of Educational Audiology, 20, 1–15.

[ref18] Knickelbein, B. A., & Richburg, C. M. (2012). Special educators’ perspectives on the services and benefits of educational audiologists. Communication Disorders Quarterly, 34(1), 17–28. 10.1177/1525740111413120.

[ref19] Luf, P. (2019). Letter to Deaf Education Programs from the Council on Education of the Deaf. https://www.ceasd.org/wp-content/uploads/2019/10/LettertoDFEDprograms.pdf.

[ref20] McInnes, S., Peters, K., Bonney, A., & Halcomb, E. (2015). An integrative review of facilitators and barriers influencing collaboration and teamwork between general practitioners and nurses working in general practice. Journal of Advanced Nursing, 71(9), 1973–1985. 10.1111/jan.12647. 25731727

[ref21] McLeskey, L., Barringer, M. D., Billingsley, B., Brownell, M., Jackson, D., Kennedy, M., Lewis, T., Maheady, L., Rodriguez, J., Scheeler, M. C., Winn, J., & Ziegler, D. (2017). High-leverage practices in special education. Arlington, VA: Council for Exceptional Children & CEEDAR Center. https://highleveragepractices.org/four-areas-practice-k-12/collaboration.

[ref22] Meyer, K. (2019). How to Advocate for Educational Audiology Kym Meyer. Audiology Online. https://www.audiologyonline.com/articles/to-advocate-for-educational-audiology-26090.

[ref23] National Association of State Directors of Special Education (2018). Optimizing Outcomes for Students who are Deaf or Hard of Hearing: Educational Service Guidelines. NASDSE. http://www.nasdse.org/docs/nasdse-3rd-ed-7-11-2019-final.pdf?fbclid=IwAR0FDGNjmLg-8mbPU1IZhOWos8dFT2f5_7ltpLpo-LvnaD0_BO4z-j9qaQ4.

[ref24] Orchard, C. A., King, G. A., Khalili, H., & Bezzina, M. B. (2012). Assessment of Interprofessional Team Collaboration Scale (AITCS): Development and Testing of the Instrument. Journal of Continuing Education in the Health Professions, 32(1), 58–67. 10.1002/chp.21123. 22447712

[ref25] Page, T. A., Harrison, M., Moeller, M. P., Oleson, J., Arenas, R. M., & Spratford, M. (2018). Service Provision for Children Who Are Hard of Hearing at Preschool and Elementary School Ages. Language, Speech, and Hearing Services in Schools, 49(4), 965–981. 10.1044/2018_LSHSS-17-0145. 30286245 PMC6430501

[ref26] Richards, K. A. R., & Hemphill, M. A. (2018). A Practical Guide to Collaborative Qualitative Data Analysis. Journal of Teaching in Physical Education, 37(2), 225–231. 10.1123/jtpe.2017-0084.

[ref26a] Richburg, C. M., & Knickelbein, B. A. (2011). Educational Audiologists: Their Access, Benefit, and Collaborative Assistance to Speech-Language Pathologists in Schools. Language, Speech, and Hearing Services in Schools, 42(4), 444–460. 10.1044/0161-1461(2011/10-0011). 21844401

[ref27] Ronfeldt, M., Farmer, S. O., McQueen, K., & Grissom, J. A. (2015). Teacher collaboration in instructional teams and student achievement. American Educational Research Journal, 52(3), 475–514. 10.3102/0002831215585562.

[ref28] Stein, R. B., & Short, P. M. (2001). Collaboration in Delivering Higher Education Programs: Barriers and Challenges. The Review of Higher Education, 24(4), 417–435. 10.1353/rhe.2001.0010.

[ref29] Wei, H., Horns, P., Sears, S. F., Huang, K., Smith, C. M., & Wei, T. L. (2022). A systematic meta-review of systematic reviews about interprofessional collaboration: Facilitators, barriers, and outcomes. Journal of Interprofessional Care, 36(5), 735–749. 10.1080/13561820.2021.1973975. 35129041

